# Comparison of *BRAF* Mutation Screening Strategies in a Large Real-Life Series of Advanced Melanoma Patients

**DOI:** 10.3390/jcm9082430

**Published:** 2020-07-30

**Authors:** Maria Colombino, Carla Rozzo, Panagiotis Paliogiannis, Milena Casula, Antonella Manca, Valentina Doneddu, Maria Antonietta Fedeli, Maria Cristina Sini, Grazia Palomba, Marina Pisano, Paolo A. Ascierto, Corrado Caracò, Amelia Lissia, Antonio Cossu, Giuseppe Palmieri

**Affiliations:** 1 Unit of Cancer Genetics, Institute of Biomolecular Chemistry (ICB), 07100 Sassari, Italy; colombinom@yahoo.it (M.C.); casulam@yahoo.it (M.C.); mariacristina.sini@cnr.it (M.C.S.); graziap68@yahoo.it (G.P.); 2 Unit of Cancer Genetics, Institute of Genetic and Biomedical Research (IRGB), National Research Council (CNR), Traversa La Crucca 3, 07100 Sassari, Italy; carlamaria.rozzo@cnr.it (C.R.); antonella.manca@cnr.it (A.M.); marina.pisano@cnr.it (M.P.); 3 Department of Medical, Surgical, and Experimental Sciences, University of Sassari, Viale San Pietro 43, 07100 Sassari, Italy; panospaliogiannis@gmail.com (P.P.); valentinadoneddu@gmail.com (V.D.); mariaant.fedeli@hotmail.it (M.A.F.); a_lissia@yahoo.it (A.L.); cossu@uniss.it (A.C.); 4 Unità Melanoma, Istituto Nazionale Tumori “Fondazione Pascale”, Via Mariano Semmola 53, 80131 Naples; Italy; paolo.ascierto@gmail.com (P.A.A.); corrado.caraco@istitutotumori.na.it (C.C.)

**Keywords:** melanoma, targeted therapies, BRAF, druggable mutations, real-time PCR, NGS assay

## Abstract

Malignant melanoma (MM) is one of the deadliest skin cancers. *BRAF* mutation status plays a predominant role in the management of MM patients. The aim of this study was to compare *BRAF* mutational testing performed by conventional nucleotide sequencing approaches with either real-time polymerase chain reaction (rtPCR) or next-generation sequencing (NGS) assays in a real-life, hospital-based series of advanced MM patients. Consecutive patients with AJCC (American Joint Committee on Cancer) stage IIIC and IV MM from Sardinia, Italy, who were referred for molecular testing, were enrolled into the study. Initial screening was performed to assess the mutational status of the *BRAF* and *NRAS* genes, using the conventional methodologies recognized by the nationwide guidelines, at the time of the molecular classification, required by clinicians: at the beginning, Sanger-based sequencing (SS) and, after, pyrosequencing. The present study was then focused on *BRAF* mutation detecting approaches only. *BRAF* wild-type cases with available tissue and adequate DNA were further tested with rtPCR (Idylla™) and NGS assays. Globally, 319 patients were included in the study; pathogenic *BRAF* mutations were found in 144 (45.1%) cases examined with initial screening. The rtPCR detected 11 (16.2%) and 3 (4.8%) additional *BRAF* mutations after SS and pyrosequencing, respectively. NGS detected one additional *BRAF*-mutated case (2.1%) among 48 wild-type cases previously tested with pyrosequencing and rtPCR. Our study evidenced that rtPCR and NGS were able to detect additional *BRAF* mutant cases in comparison with conventional sequencing methods; therefore, we argue for the preferential utilization of the aforementioned assays (NGS and rtPCR) in clinical practice, to eradicate false-negative cases and improve the accuracy of *BRAF* detection.

## 1. Introduction

Malignant melanoma (MM) is the deadliest form of skin cancer; it is estimated to affect more than 100,000 individuals, causing approximately 7000 deaths in the United States (US) in 2020 [1]. In Italy, an increase in MM incidence has been observed in the last decade, and approximately 12,300 new cases have been estimated (4% of all tumors) in 2019, representing the second and third most frequent neoplasia (9% and 7% of all tumors) in young men and women, respectively [2,3]. In the past, few effective therapeutic options were available for advanced MM patients, with response rates to conventional chemotherapy and immunomodulation therapy being limited to about 15–19% [4,5]. In the last few years, new therapeutic options have revolutionized the treatments of patients with III/IV American Joint Committee on Cancer (AJCC) stage melanomas. They include therapies targeted to specific genetic tumor mutations, as well as immunotherapy with immune checkpoint inhibitors (ICIs) [5,6]. 

Targeted therapies essentially consist of inhibitors of BRAF, a serine-threonine kinase that is constitutively activated in about one half of MMs carrying a mutation in the codon 600 of the *BRA*F oncogene [7]. Mutation at the codon V600 of the *BRAF* gene represents more than 97% of all *BRAF* mutations [8]. The most frequent alteration, occurring in about 75% of the cases, is a transversion of T to A at nucleotide 1799, which results in a substitution of a valine for glutamic acid at position 600 of the BRAF kinase (V600E) [8]. Less common substitutions are valine for lysine (V600K, up to 20%), arginine (V600R, 1%), leucine (V600M, 0,3%), and aspartic acid (V600D, 0,1%), as well as rarer mutations in other codons such as K601E or D594N [7,9]. The discovery and description of the crystal structure of the mutated BRAF protein [10] led to the development of several specific inhibitors, such as vemurafenib, dabrafenib, and encorafenib, which have been approved both in the US and Europe for the treatment of advanced (AJCC stage IIIC and stage IV) MM-harboring BRAF V600 mutations [11,12,13,14]. These BRAF inhibitors, administered in combination with MEK inhibitors, resulted in rapid therapeutic responses, with significant improvements in both progression-free and overall survival in large fractions of metastatic MM patients [2,15]. 

Molecular testing to determine *BRAF* mutation status has therefore become standard-of-care in the modern clinical management of patients with advanced MM, being currently the only available biomarker that can predict therapeutic responses to treatments with combined BRAF and MEK inhibitors. *BRAF* testing is currently recommended by both the National Comprehensive Cancer Network (NCCN) and the European Society for Medical Oncology (ESMO) guidelines for advanced melanoma patients [16,17]; the *BRAF* V600 mutation must be detected by using an FDA-approved (USA) or CE-IVD-certified (Europe) test [17,18,19]. 

More recently, several studies have demonstrated the impact of immune checkpoint inhibitors and targeted therapies on disease control in the adjuvant setting [20]. A long-term benefit of a 12-month adjuvant treatment with a combination of BRAF and MEK inhibitors (dabrafenib and trametinib, respectively) has been observed in patients with resected stage III *BRAF* V600 mutant melanoma [21,22]; in a recent update, median relapse-free survival was not reached in treated patients after a median follow-up of 60 months [23]. These findings highlight the significance of the assessment of the *BRAF* mutational status in all stage III MM patients for a more appropriate clinical decision. Overall, the detection of the *BRAF* V600 mutation plays a predictive role in the management of MM patients, identifying those with a potential sensitivity to combined treatments with BRAF and MEK inhibitors, either in advanced (stage IV or unresectable stage III) or resectable high-stage disease [24,25]. Identification of *NRAS* mutations may be also useful for a comprehensive molecular classification of the MM patients, as well as for their potential enrolment in clinical trials testing specific pharmacologic agents. 

From a clinical perspective, it is therefore mandatory to identify the best technique to detect *BRAF* mutations with the highest sensitivity and specificity. Generally, formalin-fixed and paraffin-embedded (FFPE) tissues from MM patients are used for mutational analysis, after paraffin removal and genomic DNA purification with standardized protocols. The aim of this study was to compare *BRAF* mutational testing performed with conventional nucleotide sequencing approaches (Sanger-based sequencing and pyrosequencing) and either real-time polymerase chain reaction (rtPCR) or next-generation sequencing (NGS) assays, in order to assess the levels of concordance between different techniques in a real-life, hospital-based series of 319 FFPE tissue samples from advanced MM patients from Sardinia, Italy. 

## 2. Materials and Methods

### 2.1. Patients and Samples

Patients with a histologically proven diagnosis of advanced MM (AJCC stages IIIC and IV), originating from Sardinia, Italy, were consecutively collected at clinics across the entire island and referred for molecular testing at the laboratory of the National Research Council (CNR), Sassari, Italy, from October 2012 through September 2019. All patients regularly participating in the diagnosis and treatment programs for melanoma at the Hospitals across Sardinia Island had tumor-tissue samples available for molecular analysis before inclusion in the study. To avoid bias, patients were included regardless of age of onset, family history of cancer, disease characteristics, and gene tested (*BRAF* with or without *NRAS*); their demographic, clinical, and pathological data were retrieved and stored in a digital database. FFPE tumor samples of primary melanomas or metastases from each patient were collected. Histological classification, including Breslow thickness and disease stage at diagnosis, according with the 8^th^ versions of AJCC staging system, was performed in all cases. All histological specimens with an ascertained tumor cell content greater than 60% were selected for mutation analysis; in some cases, the tissue sections were macrodissected by removing surrounding healthy tissue in order to obtain tumor samples with at least 70% neoplastic cells. All samples included in the study were assessed for the quality of the purified DNA, in order to ensure that discrepant cases could not arise from technical problems due to the insufficient sample quality.

The patients were informed about the aims and methods of the study and, before the tissue sample was collected (thus, at the time of initial molecular testing), given a written informed consent for both mutational analyses with molecular diagnostic purposes on tissue samples and participation in the study. The study was conducted in accordance with the principles of the Declaration of Helsinki and approved by the Committee for the Ethics of the Research and Bioethics of the National Research Council.

### 2.2. Molecular Testing

#### 2.2.1. DNA Isolation and Screening

Genomic DNA was isolated from tissue sections, using a standard protocol. In particular, paraffin was removed from FFPE samples with Bio-Clear (Bio-Optica, Milan, Italy), and DNA was purified by using the QIAamp DNA FFPE Tissue kit (Qiagen Inc., Valencia, CA, USA). DNA quantitation and quality assessment were carried out with both a Nanodrop 2000 spectrophotometer (Thermo Scientific, Wilmington, DE, USA) and Qubit^®^ 2.0 Fluorometer (Invitrogen, Carlsbad, CA, USA). DNA fragmentation status was evaluated with the Agilent 2200 TapeStation, system using the Genomic DNA ScreenTape assay (Agilent Technologies, Santa Clara, CA, USA), which is able to produce a DNA Integrity Number (DIN). 

#### 2.2.2. Sanger Sequencing (SS)

Polymerase chain reaction (PCR) was performed on 20 ng of genomic DNA in a Veriti 96-Well Fast Thermal Cycler (Life Technologies-Thermo Fisher Scientific, Waltham, MA, USA); all PCR-amplified products were directly sequenced using an automated ABI3130 fluorescence-cycle sequencer (Life Technologies-Thermo Fisher Scientific, Waltham, MA, USA). Sequencing conditions, as well as primer sets and PCR assay protocols, were as previously described [26,27]. Sequencing analysis was conducted in all samples in duplicate and in both directions (forward and reverse). A nucleotide sequence was considered as valid when the quality value (QV) was higher than 20 (<1/100 error probability); in this study, the QV average was 35 (range, 30–45; <1/1000–1/10,000 error probability). Starting from the purified DNA, the flat cost of the SS analysis was around €50, and the time required for performing it was about 6 h.

#### 2.2.3. Pyrosequencing

Quantitative measurements of *BRAF* mutations were performed with the Therascreen™ BRAF Pyro Kit (Qiagen Inc., Valencia, CA, USA), for the quantitative detection of mutations in codons 600, 469, and 464 of the human *BRAF* gene in genomic DNA. In particular, the Therascreen™ BRAF Pyro Kit detected the following variants: V600E (c.1799T > A/c.1799_1800TG > AA), V600G (c.1799T > G), V600A (c.1799T > C), V600M (c.1798G > A), V600D (c.1799_1800TG > AT), V600K (c.1798_1799GT > AA), V600R (c.1798_1799GT > AG), G469E (c.1406G > A), G469A (c.1406G > C), G469V (c.1406G > T), G469S (c.1405_1406GG > TC), G466E (c.1397G > A), G466V (c.1397G > T), G464E (c.1391G > A), and G464V (c.1391G > T). Each pyrosequencing assay, which included a positive (*BRAF* mutated) and a negative (*BRAF* wild-type) DNA sample as control, was performed on a PyroMark Q24 system (Qiagen Inc., Valencia, CA, USA), following the manufacturer’s instructions. Starting from the purified DNA, the flat cost of the pyrosequencing analysis was around €90, and the time required for performing was about 4 h.

#### 2.2.4. Real-Time PCR (rtPCR) Test 

The rtPCR test was based on the use of the Idylla^TM^ *BRAF* mutation assay (Biocartis, Mechelen, Belgium), a fully automated rtPCR-based diagnostic mutation analysis method. The test consists of allele-specific PCR reactions that enable the qualitative detection of the wild-type sequence (c.1799T) and the main mutations at codon 600 in *BRAF* gene: V600E (c.1799T > A), V600E2 (c.1799_1800 delinsAA), V600D (c.1799_1800 delinsAT/c.1799_1800 delinsAC), V600K (c.1798_1799 delinsAA), V600M (c.1798G > A), and V600R (c.1798_1799 delinsAG). In each rtPCR assay, an internal control to test quality of amplification is included. A solution with at least 40 nanograms of isolated genomic DNA was loaded onto the cartridge in our experiments. Starting from the purified DNA, the flat cost of the Idylla^TM^ test was around €170, and the time required for performing was about 2 h.

#### 2.2.5. Next-Generation Sequencing

Next-generation sequencing (NGS) analysis was performed by using either the Ion Torrent PGM System either the Ion S5 GeneStudio platform with a multiple-gene panel or the Oncomine Focus Assay (Life Technologies-Thermo Fisher Scientific, Waltham, MA, USA), arranged in two primer pools, and designed to explore the mutational status of selected regions within the main 52 genes involved in tumorigenesis. Amplicon libraries were generated by starting from 20 ng of genomic DNA, using the Ion AmpliSeq Library Kit-2.0 (Life Technologies-Thermo Fisher Scientific), and barcoding each sample, following the manufacturer’s instructions. Cycling conditions were performed according to the DNA type and primer pairs per pool. Libraries were purified with Agencourt Ampure-XT Beads (Beckman Coulter, Brea, CA, USA); purified DNA was diluted at a final concentration of 50 pM, placed into the Ion Chef for emulsion PCR and Chip loading, and sequenced on the Ion S5 GeneStudio (Life Technologies-Thermo Fisher Scientific) with the Ion Hi-Q™ View Sequencing Kit (Life Technologies-Thermo Fisher Scientific). Sequencing data were processed with the Ion Torrent Software Suite *v*.5.10.1 (Life Technologies-Thermo Fisher Scientific) platform-specific pipeline software. The plugin Variant Caller (VC) Ion Reporter v.5.10.1.20 and the Integrative Genome Viewer (http://www.broadinstitute.org/igv) were used for variant annotation and reads visualizations, respectively. To get a total amount of at least 10 mutated alleles for each candidate amplicon, the following mutation selection criteria were adopted: coverage of >200 reads and frequency of mutated alleles >5% for gene amplicon. Starting from the purified DNA, the flat cost of the NGS analysis with the Oncomine Focus Assay was up to €450, and the time required for performing was about two and half days.

### 2.3. Statistical Analysis

Results were expressed as median values (range) and percentages. Statistical differences between groups were evaluated by using the chi-squared test or the Fisher exact test, as appropriate. Statistical significance was set at 0.005. Statistical analyses were performed by using MedCalc for Windows, version 15.4 64 bit (MedCalc Software, Ostend, Belgium).

## 3. Results

Globally, 319 advanced melanoma patients undergoing molecular analysis for diagnostic classification of the *BRAF*/*NRAS* mutational status were consecutively collected in a hospital-based manner and enrolled into the study. The median age at diagnosis was 65 years, and 183 (57%) were males. The demographic, clinical, and pathological characteristics of the patients are reported in Table 1. Patients originated from different geographical areas across Sardinia: 241 (76%) were from Sassari province in North Sardinia, and 78 (24%) were from the remaining parts of the island. Almost the entire series was composed by patients with MM (89.4%, *n* = 285); four (1.3%) cases were affected by mucosal melanoma, and 30 (9.4%) patients presented with MM metastasis from an occult primary tumor; among them, 22 (73.3%) cases had lymph node metastasis, while the remaining patients had visceral metastasis. Most lesions were distributed on the trunk (53%, *n* = 152), followed by the limbs (32%, *n* = 93) and head/neck (15%, *n* = 44). Nodular and superficial spreading melanomas were the most frequent histological variants (50% and 39%, respectively), with vast preponderance of >1 mm thick melanomas (92%). Ulcerated and not-ulcerated melanomas were similarly represented, whereas 81% of patients showed ≥1 mitosis (Table 1).

**Figure 1:**
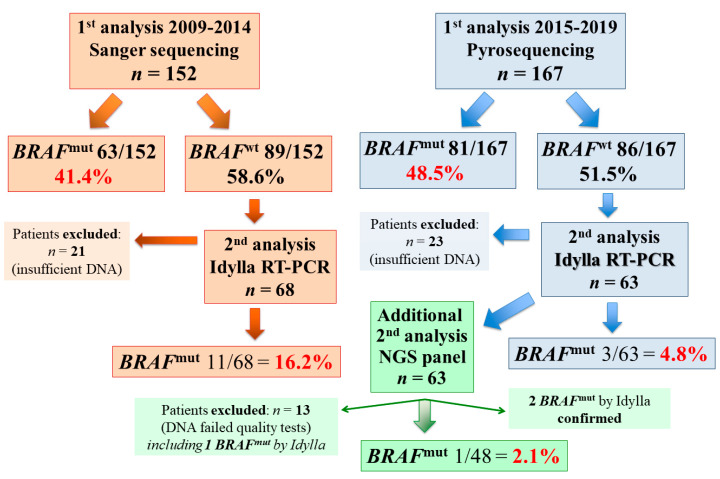
Tissue samples from advanced malignant melanoma (MM) patients analyzed for BRAF mutational status and screening methodologies used into the study. RT-PCR (written as rtPCR in the rest of the manuscript), real-time polymerase chain reaction; NGS, next-generation sequencing.

The mutational status of the *BRAF* and *NRAS* genes was initially assessed with the screening methodology conventionally used at the time of the molecular diagnosis, required by clinician for patients’ classification: Sanger-based sequencing (SS), from 2009 to 2014, and pyrosequencing, from 2015 to 2019 (Figure 1). Additional tests (Idylla^TM^ and NGS) were performed in accordance with the availability of tissue samples and adequate amount and quality of the DNA to use. *BRAF* mutations were documented in 45% (144/319) of the initial tests performed, while *NRAS* mutations were present in 15% (40/272) of the tumors tested (Table 2). No concomitant mutation in *BRAF* and *N*RAS** genes was detected, further confirming that deleterious mutations in these driver oncogenes are mutually exclusive in melanoma [28,29]. Globally, about two-thirds (184 cases) of Sardinian melanoma patients were found to carry a *BRAF* or *NRAS* mutation, even considering that some cases were not analyzed for *NRAS* mutations. No statistically significant differences have been found between genders in the distribution of mutations in both genes; however, the amount of patients aged less than 55 years was significantly higher in patients with *BRAF* mutations than in those without, and the amount of patients older than 55 was significantly greater in patients with *NRAS* mutations than in those without (Table 2). Indeed, in patients with less than 55 years of age, mutation rates were significantly higher for the *BRAF* than for the *NRAS* gene (58/95, 61% vs. 4/77, 5.2%; *p* < 0.0001). Overall, no differences in distribution of *BRAF* and *NRAS* mutations between rural and urban areas, both globally and within the two (north vs. middle-south) geographical parts of the island, were observed.

The subtypes of the mutations found for each gene are summarized in Table 3. A very high proportion of *BRAF* mutations across samples was represented by the BRAF^V600E^ variant (120, 83.3%). All but one of the remaining *BRAF* variants were represented by other V600 subtypes: V600K (19, 13.2%), V600D (3, 2.1%), and V600R (1, 0.7%) (Table 3). The K601E mutation was the only variant not affecting the codon 600 of *BRAF*, though it is a sequence variation still localized in the active kinase domain of the gene, which can respond to the targeted therapy. For *NRAS*, nearly all (35/40; 87.5%) mutations were found at the codon 61 of the gene: Q61R (*n* = 19), Q61K (*n* = 12), Q61L (*n* = 3), and Q61H (*n* = 1) (Table 3). 

All mutations detected in this study have been reported in the Human Gene Mutation Database (HGMD), at http://www.hgmd.cf.ac.uk/ac/index.php, and in the Catalogue of Somatic Mutations in Cancer (COSMIC), at http://www.sanger.ac.uk/genetics/CGP/cosmic/.

Successively, research was aimed at investigating differences in terms of sensitivity and specificity among the currently available molecular platforms for improving assessment of the mutational status. Given the high number of cases, we concentrate on *BRAF* mutations only in order to perform comparisons between the testing methods. Additional analyses with rtPCR/Idylla^TM^ and NGS assays were performed on available FFPE tissue sections and/or DNA samples deriving from the same specimens used for first *BRAF* classification. Among the 144 patients carrying a *BRAF* mutated melanoma in our series (Figure 1), 61 DNA samples (30 cases evaluated by SS assay and 31 by pyrosequencing) were selected as adequate for further analyses. The Idylla^TM^ test confirmed the presence of *BRAF* mutations in all 30 (100%) positive cases assessed by Sanger-based sequencing and in 30/31 (96.8%) mutated cases assessed by pyrosequencing. The latter discrepant case was further investigated with the NGS assay, which confirmed the presence of the *BRAF* mutation (thus confirming the result obtained by the pyrosequencing analysis).

Among the 175 *BRAF* wild-type cases, 42 of them were excluded, since the remaining available DNA was not sufficient or qualitatively adequate to perform further assays, and there was no availability of additional FFPE tissue sections (Figure 1). Overall, 131 *BRAF* wild-type samples (68 after SS and 63 after pyrosequencing) were re-analyzed by rtPCR/Idylla^TM^ test; among the formers, 11 (16.2%) additional *BRAF* mutated cases were found, while among those initially tested with pyrosequencing, 3 (4.8%) additional *BRAF* mutated cases were detected (Figure 1). The difference was statistically significant (*p* = 0.047), even if the compared cases were not the same. Finally, 48 samples out of the 63 that underwent rtPCR/Idylla^TM^ testing after pyrosequencing were also evaluated by NGS, and one additional mutation was detected; in this case, no statistically significant difference was found between the two methods (*p* = 0.637).

## 4. Discussion

*BRAF* molecular testing is currently imperative for the classification of stages III and IV MM patients, toward the selection of the appropriate therapeutic strategy. Several methods for *BRAF* testing are currently being used, including both companion diagnostic and laboratory-developed methods. The ideal method should be highly sensitive in detecting mutant alleles, and, at the same time, highly specific in detecting the correct mutation. Furthermore, it should be performable by using a small amount of biological material, considering that often the samples are small FFPE biopsies, and more than one analysis has to be carried out. In addition, the test should be inexpensive, easy, and quick to perform, with results that are easy to interpret. Currently, a unique test that contains all such features does not exist, and, often, more than one test is recommended. 

The detection of *BRAF* mutations is commonly performed on DNA extracted from tumor tissue samples, using a molecular approach. In recent years, a *BRAF* mutation analysis conducted at protein level was introduced into the clinical practice, though its use remains controversial. The protein-based test is represented by an immunohistochemistry assay with a monoclonal antibody (VE1), which is specific for detecting the expression of BRAF^V600E^ mutated protein in tumor tissue samples [30]. Nearly all tests currently in use are DNA-based. They include allele-specific PCR assays to selectively amplify the candidate *BRAF* codon and direct sequencing strategies (SS, pyrosequencing, or NGS) to determine the nucleotide sequence of the gene [31]. In Europe, at least three allele specific PCR tests have been CE-IVD certified for diagnostics: the PNAClamp™ BRAF Mutation detection kit (Panagene), the Cobas^®^ 4800 BRAF V600 mutation kit (Roche Diagnostics), and the Idylla^TM^ BRAF mutation kit (Biocartis). All rtPCR tests show higher sensitivity and specificity than those based on direct sequencing. Unfortunately, few studies have fully compared such different strategies for *BRAF* mutation screening. To date, no consensus on the best method to use in clinical practice exists. 

The Idylla^TM^ system, a fully automated rtPCR platform with several advantages over other techniques, has been recently introduced in clinical practice. Firstly, it is characterized by rapid turnaround times (approximately two hours), which is essential because it allows rapid decisions regarding the best treatment to adopt for the patient [32]. Furthermore, while the Cobas^®^ test can detect the *BRAF* V600E mutation only, the Idylla^TM^ test can detect all the actionable mutations at the codon V600 of the *BRAF* gene, including the less frequent V600D/K/R/M variants. In addition, cartridges that are able to detect the most relevant genetic alterations of both the *BRAF* and *NRAS* genes in a single assay are now available, and they can be also used in other tumors harboring such alterations (i.e., colorectal cancer) [33]. In their pivotal study, Barel et al. compared Idylla™ (*NRAS-BRAF-EGFRS492R* mutation assay—110 min per sample) with NGS and IHC for detection of *BRAF* and *NRAS* mutations in 36 patients with metastatic melanomas and found a global concordance between NGS and Idylla^TM^ assays of 97.2% (35/36 cases) [34]. Interestingly, they noticed one difference in mutation genotyping, since NGS highlighted an NRAS G13C mutation, whereas the Idylla™ cartridges, which do not search the G13C alteration, reported an NRAS G12A [34]. 

Concordance with IHC was better evaluated in the study performed by Vallèe et al. [32]. When compared with their reference, the authors found an overall concordance of 89% for BRAF V600E mutation detection by IHC, while the Idylla^TM^ system showed a concordance of 100% and 92.1% for *BRAF* and *NRAS* mutation detection, respectively. Furthermore, the Idylla^TM^ showed a PPV and NPV of both 100% for *BRAF* mutation detection and a PPV and NPV of 100% and 87%, respectively, for *NRAS* mutation detection. They concluded that BRAF V600E immunohistochemistry is efficient for detecting the V600E mutation, but negative cases should be further evaluated by molecular approaches for other BRAF mutations. 

In our study, we compared, for the first time, Idylla^TM^ with all the available sequencing techniques (Sanger-based sequencing, pyrosequencing, and NGS sequencing) in a tissue-sample collection from MM patients undergoing mutational classification for clinical purposes in a real-life, hospital-based recruitment. NGS can provide information on a wider spectrum of genetic alterations, allowing for a better evaluation of the molecular landscape of the disease; in addition, it requires limited quantities of DNA and presents the highest diagnostic sensitivity (detection limit of 1–2%) [35]. Although it is able to identify all mutations present in the analyzed genomic regions (specificity of 100%; referred to as a comprehensive test), the interpretation of sequencing data may be somehow complex and requires a high level of expertise, making its application more difficult to be broadly introduced into the clinical practice [35]. In addition, it involves a longer turnaround time, it is more expensive, and it can be affected by DNA quality. 

Our data showed that rtPCR is more accurate than both Sanger sequencing and pyrosequencing in detecting *BRAF* mutations. Firstly, we verified the existence of a quite absolute concordance among the three screening methods for the *BRAF* mutated cases analyzed in our series; overall, 60/61 (98.4%) Idylla^TM^ tests confirmed the presence of the same *BRAF* mutation identified by the sequencing assay. One could speculate that the probability of detecting a false-positive *BRAF* mutated case is rare when using one of the three different approaches described above for mutation screening. Slightly more complex is the evaluation of the data among the *BRAF* wild-type cases from our series. As expected, the Idylla^TM^ detection rates of missed *BRAF* mutations cases were significantly higher in wild-type cases assessed by SS, rather than in those by pyrosequencing. Indeed, Sanger-based direct DNA sequencing has the lowest diagnostic sensitivity (detection limit of 15–20%), though it is able to identify all the variants present in the analyzed genomic regions (specificity of 100%, again, referred to as a comprehensive test); pyrosequencing, instead, shows a higher sensitivity (detection limit of 5–8%) and a good mutation coverage (specificity of 90%; referred to as a near-comprehensive test). Finally, the ability of Idylla^TM^ to detect such mutations was comparable with that of NGS, and both methods were more accurate than pyrosequencing. It has been reported that rtPCR techniques have a very high sensitivity (detection limit of 2–3%), but they can identify a limited number of mutations within specifically targeted genomic regions (specificity for each variant of up to 98%) [36,37,38]. In our study, we did not compare molecular techniques on DNA with IHC techniques on protein.

By summarizing pros and cons for each *BRAF* mutation testing strategy, we could infer the following: 

NGS provides the maximum level of specificity (100%) and sensitivity (up to 98%) in detecting all gene variants (pros), but it requires skilled personnel and has several practical drawbacks (longer time for sample preparation and running, higher cost for reagents, and lack of guarantee of being able to complete the analysis in all FFPE samples due to DNA quality limitations) (cons); 

Sanger-based direct sequencing achieves the highest specificity (100%) and can detect all sequence mutations in *BRAF* exons 11 and 15 (pros), but it presents the lowest diagnostic sensitivity (80–85%)—which requires a higher tumor cell representation into the tissue sample—and it is somehow time-consuming (cons).

Pyrosequencing is a simple-to-perform method and provides a good level of sensitivity (92–95%) (pros), but it does not achieve a complete mutation coverage specificity (up to 90%; in gene codons 600, 469, and 464 only; see Methods) (cons). 

Real-time PCR is a rapid method which achieves the same maximum level of sensitivity of NGS (up to 98%), without requiring particular skills (pro), but it is able to identify a limited number of mutations (Idylla^TM^ test: V600E/D/K/M/R, but not other V600 actionable variants, such as V600G/A—see Methods; Cobas^®^ 4800 BRAF V600 mutation test: V600E/K mutations only) (con).

Our study has some limitations, mainly the retrospective design and the heterogeneity in the compared groups, dictated by its real-life nature. On the other hand, our work has several advantages, as it is the first study to compare four different DNA-based techniques, using the same specimen and same DNA per case, in order to avoid inter-tumoral heterogeneity. Finally, the study included patients from the same genetically homogeneous population from Sardinia, an island that experienced little immigration and genetic contamination in the past decades, due to its geographical location [39,40]. 

In conclusion, our findings provide support for both NGS and Idylla^TM^ assays to be adopted as the molecular method of choice for routine assessment of *BRAF* status in MM patients and to provide guidance toward the appropriate treatment strategy. These methods improved the diagnostic accuracy of *BRAF* testing via the detection of additional *BRAF* mutations in a subset of false-negative cases previously tested with Sanger sequencing or pyrosequencing. In attendance of further confirmations in larger prospectively designed studies, the use of two sensitive molecular methods may ensure the highest level of diagnostic accuracy. 

## Figures and Tables

**Table 1:** Patient demographics and melanoma characteristics in the study population.

Characteristics	Data
Age at diagnosis, median (range)	65 (21–92)
Male gender, *n* (%)	183 (57.4)
**Primary melanoma localization (*n* = 319)**	
Limbs, *n* (%)	93 (29.2)
Head and neck, *n* (%)	44 (13.8)
Trunk, *n* (%)	152 (47.6)
Occult, *n* (%)	30 (9.4)
Lymph node metastasis, *n* (%)	22 (6.9)
Visceral metastasis, *n* (%)	8 (2.5)
**Histology (*n* = 289)**	
SSM, *n* (%)	146 (50.5)
Nodular, *n* (%)	112 (38.7)
Acral, *n* (%)	19 (6.6)
Lentigo maligna, *n* (%)	8 (2.8)
Mucosal, *n* (%)	4 (1.4)
**Breslow class (*n* = 285)**	
≤1 mm, *n* (%)	23 (8.1)
>1-≤2 mm, *n* (%)	78 (27.4)
>2-≤4 mm, *n* (%)	95 (33.3)
>4 mm, *n* (%)	89 (31.2)
**Ulceration (*n* = 248)**	
Present, *n* (%)	137 (55.2)
Absent, *n* (%)	111 (44.8)
**Mitosis number (*n* = 243)**	
<1, *n* (%)	47 (19.3)
≥1, *n* (%)	196 (80.7)
**AJCC stage at diagnosis (*n* = 285)**	
IA-IB, *n* (%)	26 (9.2)
IIA-IIB, *n* (%)	107 (37.5)
IIC, *n* (%)	28 (9.8)
III, *n* (%)	101 (35.4)
IV, *n* (%)	23 (8.1)
**Lymph node metastasis at diagnosis (*n* = 289)**	
pN0, *n* (%)	171 (59.2)
pN+, *n* (%)	118 (40.8)

SSM, superficial spreading melanoma; AJCC, American Joint Committee on Cancer.

**Table 2:** Patient mutational status at first test screening by conventional analysis. In bold, significant *p*-value correlations.

Characteristics	Mutated	Wild-Type	*p*
***BRAF* (*n* = 319)**			
Mutated, *n* (%)	144 (45.1)	175 (54.9)	
Gender, *n* (%)			
Male, *n* (%)	79 (54.9)	104 (59.4)	0.479
Female, *n* (%)	65 (45.1)	71 (40.6)	
**Age at diagnosis, *n* (%)**			
≤55 years, *n* (%)	58 (40.3)	37 (21.1)	0.003
>55 years, *n* (%)	86 (59.7)	138 (78.9)	
***NRAS* (*n* = 272)**			
Mutated, *n* (%)	40 (14.7)	232 (85.3)	
**Gender, *n* (%)**			
Male, *n* (%)	25 (62.5)	129 (55.6)	0.522
Female, *n* (%)	15 (37.5)	103 (44.4)	
**Age at diagnosis, *n* (%)**			
≤55 years, *n* (%)	4 (10)	73 (31.5)	0.004
>55 years, *n* (%)	36 (90)	159 (68.5)	

**Table 3:** *BRAF* and *NRAS* mutation spectrum. Frequencies are related to the total amount of mutated cases in *BRAF* (*n* = 144) and *NRAS* (*n* = 40) genes.

Exon	Mutation	Base Change	Amino Acid Change	Mutated Samples	%
*BRAF*					
15	V600D	1799–1800 TG>AT	Val to Asp	3	2.1
15	V600E	1799 T>A	Val to Glu	117	81.2
15	V600E	1799_1800TG>AA	Val to Glu	3	2.1
15	V600K	1798–99 GT>AA	Val to Lys	19	13.2
15	V600R	1798–99 GT>AG	Val to Arg	1	0.7
15	K601E	1790 T>G	Leu to Arg	1	0.7
*NRAS*					
2	G12A	35 G>C	Gly to Ala	1	2.5
2	G13D	38 G>A	Gly to Asp	2	5.0
2	G13R	37 G>C	Gly to Arg	2	5.0
3	Q61H	183 A>T	Gln to His	1	2.5
3	Q61K	181 C>A	Gln to Lys	12	30.0
3	Q61L	182 A>T	Gln to Leu	3	7.5
3	Q61R	182 A>G	Gln to Arg	19	47.5

## References

[1] Siegel R.L., Miller K.D., Jemal A. (2020). Cancer statistics, 2020. CA Cancer J. Clin..

[2] Cossu A., Casula M., Cesaraccio R., Lissia A., Colombino M., Sini M.C., Budroni M., Tanda F., Paliogiannis P., Palmieri G. (2017). Epidemiology and genetic susceptibility of malignant melanoma in North Sardinia, Italy. Eur. J. Cancer Prev..

[3] I Numeri del Cancro in Italia. https://www.aiom.it/wp-content/uploads/2019/09/2019_Numeri_Cancro-operatori-web.pdf.

[4] Parker B.S., Rautela J., Hertzog P.J. (2016). Antitumor actions of interferons: Implications for cancer therapy. Nat. Rev. Cancer.

[5] Luke J.J., Flaherty K.T., Ribas A., Long G.V. (2017). Targeted agents and immunotherapies: Optimizing outcomes in melanoma. Nat. Rev. Clin. Oncol..

[6] Su M.Y., Fisher D.E. (2016). Immunotherapy in the precision medicine era: Melanoma and beyond. PLoS Med..

[7] Sini M.C., Doneddu V., Paliogiannis P., Casula M., Colombino M., Manca A., Botti G., Ascierto P.A., Lissia A., Cossu A. (2018). Genetic alterations in main candidate genes during melanoma progression. Oncotarget.

[8] Davies H., Bignell G.R., Cox C., Stephens P., Edkins S., Clegg S., Teague J., Woffendin H., Garnett M.J., Bottomley W. (2002). Mutations of the BRAF gene in human cancer. Nature.

[9] Greaves W.O., Verma S., Patel K.P., Davies M.A., Barkoh B.A., Galbincea J.M., Yao H., Lazar A.J., Aldape K.D., Medeiros L.J. (2013). Frequency and spectrum of BRAF mutations in a retrospective, single institution study of 1112 cases of melanoma. J. Mol. Diagn..

[10] Wan P.T., Garnett M.J., Roe S.M., Lee S., Niculescu-Duvaz D., Good V.M., Project C.G., Jones C.M., Marshall C.J., Springer C.J. (2004). Mechanism of activation of the RAF-ERK signaling pathway by oncogenic mutations of B-RAF. Cell.

[11] Chapman P.B., Hauschild A., Robert C., Haanen J.B., Ascierto P., Larkin J., Dummer R., Garbe C., Testori A., Maio M. (2011). BRIM-3 Study Group. Improved survival with vemurafenib in melanoma with BRAF V600E mutation. N. Engl. J. Med..

[12] Hauschild A., Grob J.J., Demidov L.V., Jouary T., Gutzmer R., Millward M., Rutkowski P., Blank C.U., Miller W.H., Kaempgen E. (2012). Dabrafenib in BRAF-mutated metastatic melanoma: A multicentre, open-label, phase 3 randomised controlled trial. Lancet.

[13] Dummer R., Hauschild A., Lindenblatt N., Pentheroudakis G., Keilholz U. (2016). Cutaneous melanoma: ESMO clinical practice guidelines for diagnosis, treatment, and follow-up. Ann. Oncol..

[14] Seth R., Messersmith H., Kaur V., Kirkwood J.M., Kudchadkar R., McQuade J.L., Provenzano A., Swami U., Weber J., Alluri K.C. (2020). Systemic therapy for melanoma: ASCO Guideline. J. Clin. Oncol..

[15] Ascierto P.A., Agarwala S.S., Botti G., Budillon A., Davies M.A., Dummer R., Ernstoff M., Ferrone S., Formenti S., Gajewski T.F. (2019). Perspectives in melanoma: Meeting report from the Melanoma Bridge (November 29th-1 December 1st, 2018, Naples, Italy). J. Transl. Med..

[16] National Comprehensive Cancer Network Guidelines. https://www.nccn.org/professionals/physician_gls/default.aspx.

[17] Michielin O., van Akkooi A.C.J., Ascierto P.A., Dummer R., Keilholz U., ESMO Guidelines Committee (2019). Electronic address: Clinicalguidelines@esmo.org. Cutaneous melanoma: ESMO Clinical Practice Guidelines for diagnosis, treatment and follow-up. Ann. Oncol..

[18] Coit D.G., Thompson J.A., Albertini M.R., Barker C., Carson W.E., Contreras C., Daniels G.A., DiMaio D., Fields R.C., Fleming M.D. (2019). Cutaneous Melanoma, Version 2.2019, NCCN Clinical Practice Guidelines in Oncology. J. Natl. Compr. Cancer Netw..

[19] Garbe C., Amaral T., Peris K., Hauschild A., Arenberger P., Bastholt L., Bataille V., Del Marmol V., Dréno B., Fargnoli M.C. (2020). European consensus-based interdisciplinary guideline for melanoma. Part 2: Treatment—Update 2019. Eur. J. Cancer.

[20] Poklepovic A.S., Luke J.J. (2020). Considering adjuvant therapy for stage II melanoma. Cancer.

[21] Long G.V., Hauschild A., Santinami M., Atkinson V., Mandalà M., Chiarion-Sileni V., Larkin J., Nyakas M., Dutriaux C., Haydon A. (2017). Adjuvant Dabrafenib plus Trametinib in Stage III BRAF-Mutated Melanoma. N. Engl. J. Med..

[22] Hauschild A., Dummer R., Schadendorf D., Santinami M., Atkinson V., Mandalà M., Chiarion-Sileni V., Larkin J., Nyakas M., Dutriaux C. (2018). Longer Follow-Up Confirms Relapse-Free Survival Benefit with Adjuvant Dabrafenib Plus Trametinib in Patients with Resected BRAF V600-Mutant Stage III Melanoma. J. Clin. Oncol..

[23] Hauschild A., Dummer R., Santinami M., Atkinson V., Mandalà M., Kirkwood J.M., Chiarion Sileni V., Larkin J.M.G., Nyakas M., Dutriaux C. (2020). Long-term benefit of adjuvant dabrafenib + trametinib (D+T) in patients (pts) with resected stage III BRAF V600–mutant melanoma: Five-year analysis of COMBI-AD. J. Clin. Oncol..

[24] Dummer R., Ascierto P.A., Gogas H.J., Arance A., Mandala M., Liszkay G., Garbe C., Schadendorf D., Krajsova I., Gutzmer R. (2018). Overall survival in patients with BRAF-mutant melanoma receiving encorafenib plus binimetinib versus vemurafenib or encorafenib (COLUMBUS): A multicentre, open-label, randomised, phase 3 trial. Lancet Oncol..

[25] Ascierto P.A., Lewis K.D., Di Giacomo A.M., Demidov L., Mandalà M., Bondarenko I., Herbert C., Mackiewicz A., Rutkowski P., Guminski A. (2020). Prognostic impact of baseline tumour immune infiltrate on disease-free survival in patients with completely resected, BRAFv600 mutation-positive melanoma receiving adjuvant vemurafenib. Ann. Oncol..

[26] Colombino M., Capone M., Lissia A., Cossu A., Rubino C., De Giorgi V., Massi D., Fonsatti E., Staibano S., Nappi O. (2012). BRAF/NRAS mutation frequencies among primary tumors and metastases in patients with melanoma. J. Clin. Oncol..

[27] Colombino M., Lissia A., Franco R., Botti G., Ascierto P.A., Manca A., Sini M.C., Pisano M., Paliogiannis P., Tanda F. (2013). Unexpected distribution of cKIT and BRAF mutations among southern Italian patients with sinonasal melanoma. Dermatology.

[28] (2015). The Cancer Genome Atlas Network. Genomic classification of cutaneous melanoma. Cell.

[29] Palmieri G., Ombra M., Colombino M., Casula M., Sini M., Manca A., Paliogiannis P., Ascierto P.A., Cossu A. (2015). Multiple molecular pathways in melanomagenesis: Characterization of therapeutic targets. Front. Oncol..

[30] Feller J.K., Yang S., Mahalingam M. (2013). Immunohistochemistry with a mutation-specific monoclonal antibody as a screening tool for the BRAFV600E mutational status in primary cutaneous malignant melanoma. Mod. Pathol..

[31] Sholl L.M., Andea A., Bridge J.A., Cheng L., Davies M.A., Ehteshami M., Gangadhar T.C., Kamel-Reid S., Lazar A., Raparia K. (2016). Template for reporting results of biomarker testing of specimens from Patients with melanoma. Arch. Pathol. Lab. Med..

[32] Vallée A., Denis-Musquer M., Herbreteau G., Théoleyre S., Bossard C., Denis M.G. (2019). Prospective evaluation of two screening methods for molecular testing of metastatic melanoma: Diagnostic performance of BRAF V600E immunohistochemistry and of a NRAS-BRAF fully automated real-time PCR-based assay. PLoS ONE.

[33] Palomba G., Doneddu V., Cossu A., Paliogiannis P., Manca A., Casula M., Colombino M., Lanzillo A., Defraia E., Pazzola A. (2016). Prognostic impact of KRAS, NRAS, BRAF, and PIK3CA mutations in primary colorectal carcinomas: A population-based study. J. Transl. Med..

[34] Barel F., Guibourg B., Lambros L., Le Flahec G., Marcorelles P., Uguen A. (2018). Evaluation of a Rapid, Fully Automated Platform for Detection of BRAF and NRAS Mutations in Melanoma. Acta Derm. Venereol..

[35] Palmieri G., Colombino M., Casula M., Manca A., Mandalà M., Cossu A., Italian Melanoma Intergroup (IMI) (2018). Molecular pathways in melanomagenesis: What we learned from next-generation sequencing approaches. Curr. Oncol. Rep..

[36] Ihle M.A., Fassunke J., König K., Grünewald I., Schlaak M., Kreuzberg N., Tietze L., Schildhaus H.U., Büttner R., Merkelbach-Bruse S. (2014). Comparison of high resolution melting analysis, pyrosequencing, next gene-ration sequencing and immunohistochemistry to conventional Sanger sequencing for the detection of p.V600E and non-p.V600E BRAF mutations. BMC Cancer.

[37] Bruno W., Martinuzzi C., Andreotti V., Pastorino L., Spagnolo F., Dalmasso B., Cabiddu F., Gualco M., Ballestrero A., Bianchi-Scarrà G. (2017). Heterogeneity and frequency of BRAF mutations in primary melanoma: Comparison between molecular methods and immunohistochemistry. Oncotarget.

[38] Mosko M.J., Nakorchevsky A.A., Flores E., Metzler H., Ehrich M., van den Boom D.J., Sherwood J.L., Nygren A.O. (2016). Ultrasensitive detection of multiplexed somatic mutations using MALDI-TOF Mass Spectrometry. J. Mol. Diagn..

[39] Casula C., Muggiano A., Cossu A., Budroni M., Caracò C., Ascierto P.A., Pagani E., Stanganelli I., Canzanella S., Sini M.C. (2009). Role of key-regulator genes in melanoma susceptibility and pathogenesis among patients from South Italy. BMC Cancer.

[40] Palomba G., Loi A., Porcu E., Cossu A., Zara I., Budroni M., Dei M., Lai S., Mulas A., Olmeo N. (2015). Genome-wide association study of susceptibility loci for breast cancer in Sardinian population. BMC Cancer.

